# Cytokines in Thyroid-Associated Ophthalmopathy

**DOI:** 10.1155/2022/2528046

**Published:** 2022-11-14

**Authors:** Pengbo Zhang, Huang Zhu

**Affiliations:** Department of Ophthalmology, Xinhua Hospital Affiliated to Shanghai Jiao Tong University School of Medicine, Shanghai 200092, China

## Abstract

Thyroid-associated ophthalmopathy (TAO), also known as thyroid eye disease (TED) or Graves' orbitopathy (GO), is a complex autoimmune condition causing visual impairment, disfigurement, and harm to patients' physical and mental health. The pathogenesis of TAO has not been fully elucidated, and the mainstream view is that coantigens shared by the thyroid and orbit trigger remodeling of extraocular muscles and orbital connective tissues through an inflammatory response. In recent years, cytokines and the immune responses they mediate have been crucial in disease progression, and currently, common evidence has shown that drugs targeting cytokines, such as tocilizumab, infliximab, and adalimumab, may be novel targets for therapy. In this review, we summarize the research development of different cytokines in TAO pathogenesis in the hope of discovering new therapeutic targets.

## 1. Introduction

**Figure 1:**
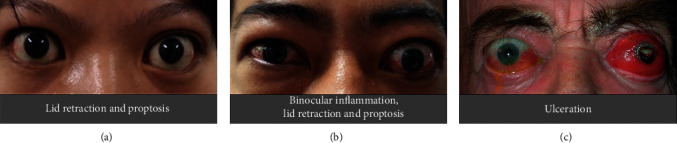
(a) Lid retraction and proptosis. (b) Binocular inflammation. (c) Ulceration. Reproduced with permission from Douglas [168].

Thyroid-associated ophthalmopathy (TAO) is a complex autoimmune orbitopathy constituting the ophthalmic manifestations of generalized thyroid-associated autoimmune disease and the most frequent extrathyroidal expression of Graves's disease (GD), which may cause double vision, edema, retracting eyelids, orbital disfigurement proptosis, and even loss of vision [1]. The quality of life and mental health of TAO patients are greatly affected by serious complications [2–4] (Figures 1(a)–1(c)). The incidence of this condition was estimated to be 16/100000 in women and 2.9/100000 in men, and the prevalence was approximately 10/10000, at least [5, 6]. Currently, identified risk factors for the development and exacerbation of TAO include stressful life events, genetics and ethnicity, sex, age, smoking, thyroid dysfunction, and thyroid-stimulating hormone receptor antibodies (TRAbs) [7, 8]. Among them, cigarette smoking is the strongest modifiable risk factor for TAO, and there is a positive correlation between risk and daily cigarette consumption [9]. The European Group on Graves' Orbitopathy (EUGOGO) classifies TAO disease severity as sight-threatening, moderate to severe, and mild and introduced one score each for clinical activity (CAS) and clinical severity (CSS) [10, 11]. The exact association of TAO and thyroid remains ambiguous: TAO was observed in 90% of patients with GD (hyperthyroid patients), but TAO may also be observed in euthyroid (5%) or hypothyroid (5%) patients [12, 13]. In this review, we used GD to describe the ophthalmic manifestations associated with autoimmune hyperthyroidism.

Cross-reactivity against antigens in both orbital and thyroid tissues is thought to be the basis of TAO pathogenesis, and a shared thyroid-specific protein within the orbit may be the initial factor [14, 15]. Orbital fibroblasts are considered predominant in the process of extensive orbital tissue remodeling via immune responses induced by autoantigens (TSHR and IGF-IR) [16–18], which cause the production of proinflammatory cytokines and the synthesis of hyaluronan [19]. The recruitment and infiltration of various immune cells into the orbit determine pathological changes in orbital tissues [20]. These cells are primary CD4+ T cells, and there are also minor populations of CD8+ cells, macrophages, plasma cells, and B cells, which focally and diffusely infiltrate the adipose tissues, extraocular and levator muscles, and lacrimal glands in active TAO [21–23]. Cytokines from immune cells led to the production of ongoing inflammation in the orbital adipose tissue and fibrous tissue of extraocular muscles, which caused increased pressure within the bony cavity [24, 25].

Importantly, no treatment has been shown to prevent or reverse the underlying pathological changes caused by TAO [15]. Active disease is usually treated with glucocorticoids, surgeries, rituximab (RTX), and orbital irradiation [26–28], which exert therapeutic effects by inhibiting the immunological process in TAO or by excising diseased tissue. Over the past decades, anticytokine therapies, such as teprotumumab, tocilizumab, infliximab, and adalimumab, have played an increasingly important role in the treatment of TAO [29]. In this review, we provide a detailed overview of the role of cytokines in TAO pathogenesis in the hope of finding new ideas for targeted cytokine therapy in TAO.

## 2. Overview of Cytokines

Cytokines are produced by immune cells, such as T cells, monocytes, B cells, NK cells, and macrophages, and certain nonimmune cells, such as fibroblasts, table dermatomes, and endothelial cells (Table 1). They are small molecule proteins that have a wide range of biological activities, can regulate the immune response, and are synthesized and secreted by stimulated immune cells [30]. Cell growth, effects, and differentiation can be regulated by the binding of cytokines and corresponding receptors [31]. Cytokines can be classified as interleukins (ILs), the tumor necrosis factor (TNF) superfamily, interferons (IFNs), colony-stimulating factors (CSFs), chemokines, and growth factors (GFs), according to structure and function. The characteristic features of cytokines are pleiotropism, redundancy, synergy, antagonism, and network. Studies have demonstrated that interleukins can be secreted not only by leukocytes but also by other types of cells, such as fibroblasts and keratinocytes, which can regulate the functional activities of other cells in addition to leukocytes. In fact, none of the cytokines are exclusively secreted by only one cell type [32, 33]. Cytokines have multiple modes of action. They can act on the same cells secreting them (autocrine), affect neighboring cells (paracrine), and act on target cells via the circulatory system [34].

### 2.1. IGF-I/IGF-IR in TAO

**Figure 2:**
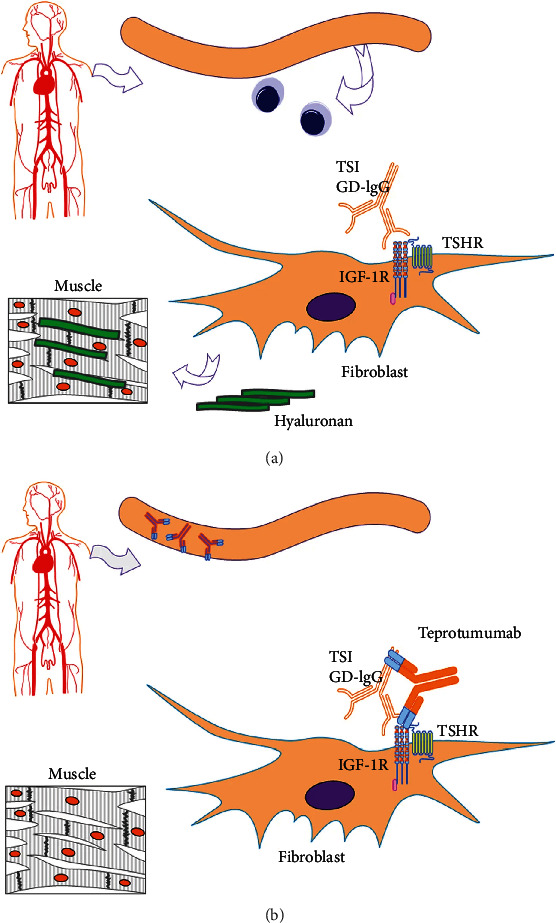
(a) Hyaluronan is produced by the binding of pathogenic autoantibodies and receptors of orbital fibroblasts and causes symptoms of thyroid eye diseases. (b) Teprotumumab blocks the binding of pathogenic autoantibodies and its receptor on orbital fibroblasts. Reproduced with permission from Douglas [168].

Insulin-like growth factor-1 (IGF-I) is vital for the regulation of mammalian development, metabolism, and growth [35]. IGF-I receptor (IGF-IR) is a membrane-spanning tyrosine kinase receptor that shares structural identity with the insulin receptor, which comprises two *α* subunits and two *β* subunits and can form heterodimers with the insulin receptor [36, 37]. Recent studies have found that the expression levels of IGF-I and IGF-IR in orbital fibroblasts of TAO were significantly increased, and IGF-IR was considered to be potentially involved in the pathogenesis of TAO [38–40]. Evidence of IGF-IR involvement in TAO was observed by Weightman et al., Rotella et al., and Perros et al. who found that IgG collected from patients with GD (GD-IgG) could displace radiolabeled IGF-I from binding sites on the surfaces of orbital fibroblasts from extraocular muscles in both TAO and non-TAO patients [41–43]. In addition, the IgG in pooled serum samples obtained from GD patients initiated the mTor/FRAP/Akt/p70s6k signaling pathway and induced the expression of the chemokine called RANTES (regulated on activation normal T-cell expressed and secreted) and IL-16 in orbital fibroblasts from TAO patients [44, 45], which play important roles in the trafficking of cells to sites of tissue destruction and remodeling [46, 47]. It is worth noting that GD-IgG could stimulate signaling pathways in TAO orbital fibroblasts but not in those cells from healthy people [44]. The induction of IL-16 and RANTES could be inhibited by specific monoclonal antibodies that block IGF-IR and 1H7 and by the transfection of a dominant negative mutant IGF-IR, 486/STOP [44], suggesting that IGF-IR signaling mediates the process. Moreover, binding of IgG and IGF-IR could induce the generation of hyaluronan in TAO orbital fibroblasts but not in normal orbital cells [48] (Figure 2(a)). Tramontano et al. reported that both IGF-I and insulin could strengthen the actions of TSH and TSIs in FRTL5 cells, which demonstrated an overlapping pathway between TSH and IGF-I [49]. Tsui et al. [50] found that IGF-IR and TSHR were colocalized on orbital fibroblasts, human thyroid epithelial cells, and orbital fat from patients with TAO. They observed that the two receptor proteins could be precipitated from solution with monoclonal antibodies against either receptor, and this study further demonstrated that signaling initiated from TSHR was dependent on IGF-IR activity to activate Erk 1/2, which is the downstream kinase of the signaling pathway. Using 1H7, an IGF-IR inhibiting antibody, could attenuate signaling introduced by rhTSH, IGF-I, and GD-IgG, which strongly suggested that IGF-IR transactivation by TSHR plays a critical role in actions mediated by TSHR [50]. Studies have shown that TSHR and IGF-IR can form functional complexes, and the two synergistically promote hyaluronan accumulation with multiple cytokines, leading to inflammation and expansion of muscle and adipose tissue [51, 52].

Teprotumumab is a recombinant monoclonal antibody of the IgG1 subclass that can bind to IGF-IR with high affinity, block the activation of IGF-IR by its endogenous ligands (IGF-I and IGF-II), and cause its internalization [52, 53]. Teprotumumab could fully block the pathophysiological responses of activation of orbital fibroblasts by autoantibodies, which leads to release of chemoattractant cytokines, T-cell infiltration, and extensive remodeling of orbital tissues [44, 54] (Figure 2(b)). A multicenter randomized clinical trial conducted by Smith et al. [54] revealed that inhibition of IGF-IR with teprotumumab was more effective than placebo in patients with moderate to severe TAO in improving CAS and proptosis, including improvements in double vision.

### 2.2. CD40/CD154 in TAO

CD40 is a costimulatory member of the tumor necrosis factor receptor (TNFR) superfamily [55]. It is trimerized after binding to CD40 ligand (CD154), and then TNF*α* receptor-associated factors (TRAFs) are recruited to the cytoplasmic domain, interfering with signal transduction pathways [56]. Activation of B lymphocytes requires stimulation and costimulation by binding of antigens and antigen receptors. CD40 is constitutively expressed on human B lymphocytes, and signaling through the binding of CD40/CD154 promotes the proliferation and activation of B cells [57]. Fries et al. [58] found that CD40 was expressed on the surface of orbital fibroblasts in TAO patients. CD154 was expressed on the surface of T lymphocytes, and CD154+ T lymphocyte infiltration was found in orbital tissues from TAO patients. In addition, soluble CD154 levels were significantly higher in the serum of TAO patients than in the normal population [59, 60]. Recently, TSHR-CD40 was shown to colocalize on the surface of fibrocytes via confocal microscopy [61]. In orbital fibroblasts, CD40-CD154 results in transcriptional activation of the endoperoxide H synthase 2 (PGHS-2) gene, hyaluronan synthesis, and production of prostaglandin E2 (PGE2), inducing proinflammatory cytokine production, including IL-6, IL-8, intercellular adhesion molecules-1 (ICAM-1), and macrophage chemoattractant protein (MCP-1) [62–64]. Increased CD40 levels were found in orbital fibroblasts, and CD40 expression in orbital fibroblasts from GD patients was enhanced when treated with TSH or IFN-*γ* [65, 66]. Gillespie et al. found that the frequency of circulating CD40+ fibrocytes, similar to the expression of CD40 in orbital fibroblasts, was obviously increased in TAO patients; signaling provoked by CD40/CD154 resulted in the production of IL-6; and IL-6 expression was mediated through the Akt and NF-*κ*B pathways [67]. Feldon et al. [68] found that autologous connate T cells could stimulate the proliferation of orbital fibroblasts in TAO patients, which was dependent on the CD40/CD156 signaling pathway, and the CD40-CD155 costimulatory pathway could mediate the activation of orbital fibroblasts from TAO patients via T lymphocytes, initiating the gene transcription of ICAM-1 [62].

Because of the important role of CD40 in the pathogenesis of TAO, the CD40 pathway has consequently become an attractive therapeutic target. Unfortunately, clinical trials targeting CD40L were halted due to adverse incidents involving thromboembolic events, which were thought to result from the crosslinking of CD154 from platelets, leading to the activation and aggregation of platelets [69, 70]. It is necessary to conduct placebo-controlled studies to fully evaluate the effectiveness and safety of targeting the CD40-CD154 pathway in TAO.

### 2.3. IL-6/IL-6R in TAO

IL-6, discovered in 1986 [71], is a pleiotropic cytokine involved in immune responses, hematopoiesis, embryonic development, inflammation, bone metabolism, and other fundamental processes [72–74]. IL-6R is composed of gp130, a signal transducer, and IL-6-binding receptor molecule (IL-6R*α*), which becomes the basis for IL-6 to exert its biological activity [75]. IL-6 promotes Th17 development in naïve T cells under the guidance of IL-23 and TGF-*β* [76] and inhibits Treg differentiation induced by TGF-*β* [77]. Studies have found that serum IL-6 levels are elevated in GD patients and are especially high in TAO patients [78–80]. The serum concentration of soluble IL-6 receptor is elevated in active TAO patients and correlates with disease activity [81]. IL-6 expression in orbital fibroblasts from GD patients and fibrocytes could be induced by stimulation of TSH and TSIs, and this process could be mediated by gene promoter activation and enhanced mRNA stability [82]. IL-6 expression is upregulated in fibrocytes by a cAMP-independent mechanism and is mediated by activation of the AKT, PKC, and PDK1 pathways by TSH [82]. Palmitate is one of the most abundant free fatty acids (FFAs) in plasma and can aggravate inflammation by promoting proinflammatory cytokines. Paik et al. found that palmitate induced the secretion of IL-6 and MCP-1 in orbital fibroblasts derived from patients with thyroid-associated ophthalmopathy and that IL-6 expression was induced by the p38, ERK, and JNK pathways [83].

Tocilizumab (TCZ) is a humanized IL-6 receptor monoclonal antibody that binds both soluble and membrane-bound receptors and is a recombinant IgG1 antibody [84]. TCZ could bind to the IL-6 binding site of membrane-bound IL-6R and sIL-6R, neutralizing IL-6-mediated activities [85]. TCZ has shown promise in treating various immune disorders, including giant cell arteritis, amyloidosis, systemic lupus erythematosus (SLE), relapsing polychondritis, and rheumatoid arthritis (RA) [86, 87]. There is a case report showing that TAO patients who are intolerant of glucocorticoid therapy and whose conventional treatment is not effective have decreased CAS scores and significantly improved eye symptoms after treatment with TCZ [88]. A randomized controlled trial conducted by Perez-Moreiras et al. enrolled 32 glucocorticoid-resistant patients with moderate to severe TAO who were administered TCZ or placebo, and the primary outcome showed that a reduction of at least 2 points in CAS at week 16 was present in 93.3% of patients, which was significantly higher than the placebo group at 58.8% [89]. Overall, in patients with active TAO that is not sensitive to hormones, TCZ may reduce the CAS score and improve symptoms of proptosis, eye movement disturbance, and diplopia or have the effect of blocking the inflammatory cascade [90, 91].

### 2.4. TNF-*α* in TAO

TNF-*α* is a cytokine that has pleiotropic effects on various cell types; it has been identified as a major regulator of inflammatory responses and is known to be involved in the pathogenesis of some autoimmune diseases [92]. TNF-*α* exists in a soluble and transmembrane form with a homotrimeric protein structure consisting of 157 amino acids, and it is generated by T lymphocytes, macrophages, and natural killer cells [93]. A meta-analysis including ten case-control studies evaluated rs1800629 and rs361525 in the TNF-*α* gene in the susceptibility of GD patients, and the data showed that an increased risk of GD was associated with the promoter SNP rs1800629 in the TNF-*α* gene [94]. A study showed that polymorphisms of the 5′ flanking region of the TNF-*α* gene at position (-1031T/C, -863C/A) are positively correlated with the development and severity of TAO [95]. Interestingly, a significant correlation between -863C/A and TAO was demonstrated by Yang et al. [96]; however, a study in Polish patients showed no significant association of this locus with TAO, and another locus (-283G/A) was significantly correlated with TAO [97]. The protein and mRNA of TNF-*α* appear to be overexpressed in orbital connective tissue in TAO patients [98, 99]. TNF-*α* participates in the inflammation and remodeling of the orbital tissues of TAO in several ways. TNF-*α* can induce ICAM-1 expression in orbital fibroblasts [100], which can promote the recruitment of inflammatory cells and exacerbate the progression of inflammation. TNF-*α* has been shown to increase the production of glycosaminoglycans (GAGs) in orbital fibroblasts [101], which causes consequent orbital tissue volume expansion in active TAO. In addition to increased GAG production, one of the main mechanisms of orbital tissue expansion is adipogenesis, and TNF-*α* has an inhibitory effect on adipogenesis in orbital fibroblasts [102], which can suppress the worsening of TAO caused by increased orbital fat. Knocking down SLIT2, an axon guidance glycoprotein, in orbital fibroblasts of GD patients upregulated the expression of TNF-*α* and IL-6 [103], which may represent an attractive therapeutic target for TAO.

Given the important contribution of TNF-*α* to the pathogenesis of TAO, regulating TNF-*α* has been considered an option to treat TAO. Adalimumab is an IgG1 monoclonal antibody whose function and structure are identical to that of natural human IgG1 and is capable of specifically blocking the binding of human TNF-*α* to receptors [104]. In a retrospective study conducted by Ayabe et al. [105], 10 patients with TAO were treated with adalimumab for 12 weeks, and in six patients, the periorbital inflammatory score decreased, suggesting that adalimumab exerts anti-inflammatory effects to improve the symptoms and signs of TAO. Infliximab, a recombinant chimeric monoclonal antibody blocking the binding of TNF-*α* to its receptors, whose effectiveness has been demonstrated in three cases of severe steroid- and surgical-resistant TAO, achieved complete remission after interval administration [106–108]. In addition, a single dose of infliximab resulted in a reduction in inflammation and improvement of visual function with no short-term side effects [109]. Etanercept is a dimeric protein able to bind to sTNF-*α* or tmTNF-*α* and inactivate them by blocking the interaction with receptors [110]. In a study conducted by Paridaens et al. [111], 10 patients with mildly to moderately active TAO were treated with etanercept, and 60% of patients were significantly improved with no serious adverse events over a mean follow-up of 18 months. In one patient with TAO associated with primary hypothyroidism and rheumatoid arthritis (RA), improvement in ocular symptoms and proptosis was observed after 4 months of etanercept administration [112]. Overall, TNF-*α* inhibitors may improve clinical symptoms and reduce the periorbital inflammatory response in patients with TAO, but randomized controlled trials are needed to further evaluate their efficacy.

### 2.5. IL-1 Superfamily in TAO

There are 11 members in the IL-1 superfamily, which can be divided into anti-inflammatory cytokines (IL-1 receptor antagonist (IL-1Ra), IL-37, IL-36Ra, and IL-38) and proinflammatory cytokines (IL-1*α*, IL-1*β*, IL-33, IL-18, and IL-36*α*, *β*, and *γ*) [113].

IL-1 was one of the first cytokines to be discovered, and it is mainly produced by monocytes, T lymphocytes, macrophages, B lymphocytes, and NK cells, in addition to being produced by almost all nucleated cells [114]. IL-1*α* and IL-1*β* are proinflammatory cytokines involved in acute and chronic inflammatory responses, and they share the same receptor complex, IL-1R [115]. Serum IL-1*α* levels in normal subjects versus patients with TAO, before versus after treatment with hormones, and in active TAO versus remission were significantly different, but only a few studies reported significant associations between genetic polymorphisms and TAO [116]. There were significant differences in the expression and regulation of the IL-1Ra gene between orbital fibroblasts from TAO patients and healthy controls. For example, the amount of intracellular soluble IL-1Ra expressed and released by retrobulbar fibroblasts derived from TAO patients was significantly lower than that from healthy controls [117]. A study conducted by Khalilzadeh et al. [118] found that there was a significant correlation between gene polymorphism and TAO, and the results of IL-1*β* are controversial, with some studies considering both negative [119] and some considering both positive [120]. A meta-analysis showed that rs1800587 (IL-1*α*, -889T/C) and rs16944 (IL-1*β*, -511A/G) polymorphisms resulted in susceptibility to TAO in an Asian population [120]. Li and Smith found that TSH induces the expression of IL-1Ra in fibrocytes and orbital fibroblasts from patients with Grave's disease [121], which directly links the activities of the TSH and IL-1 pathways for the first time. Strong responses of TAO fibroblasts to IL-1*β* are a consequence of lower levels of secreted IL-1Ra (sIL-1Ra) compared with fibrocytes, and the high levels of sIL-1Ra in fibrocytes are diminished as these cells transition to orbital fibroblasts, which provides a potential target for disease susceptibility [122]. Human orbital fibroblasts can express high levels of IL-6 when treated with IL-1*β*, whose magnitude is considerably greater than that in dermal fibroblasts and involves upregulation of IL-6 mRNA levels. This upregulation enhanced IL-6 gene promoter activity and blocked IL-6 mRNA decay by IL-1*β* [123]. In the same year, Han and Smith [124] found that tissue inhibitor of metalloproteinases-1 (TIMP-1) in human orbital fibroblasts can be induced by IL-1*β* and that treatment with IL-4 or IFN-*γ* could block IL-1*β* induction by attenuating TIMP-1 gene promoter activity.

IL-38 was discovered in 2001 and can be produced by various cells, such as keratinocytes, peripheral blood mononuclear cells (PBMCs), fibroblast-like synovial cells, and immune cells [125, 126]. IL-38 was initially considered a typical antagonist of IL-1Ra, IL-36Ra, and IL-37, but interestingly, Boutet et al. [127] found that IL-38 had an anti-inflammatory effect only at high concentrations. Therefore, the function of IL-38 is controversial. Recently, a study showed that IL-38 levels decreased in the orbital connective tissues and in the circulation of TAO patients compared with healthy controls, and the levels were significantly correlated with CAS. In addition, IL-38 exerted powerful anti-inflammatory and antifibrotic effects in vitro [128]. Pan et al. found that increased IL-38 could not only inhibit the expression of IL-23R and IL-17A in PBMCs but also suppress inflammation in orbital fibroblasts in TAO patients, and IL-38 could be a potential therapeutic approach for TAO [129].

IL-18 is a powerful proinflammatory cytokine that modulates both the innate and adaptive immune systems, enhances chemokine and cytokine production, and stimulates chemotaxis of neutrophils and lymphocytes and IFN-*γ* production by NK cells [130–132]. A study conducted by Myśliwiec et al. [133] showed that serum levels of IL-18 in TAO and GD patients were significantly higher than those in control groups, and after treatment with corticosteroids, the serum levels of IL-18 were significantly decreased when compared to those in pretreatment groups. However, in another study, researchers found that serum IL-18 levels were not increased in patients with untreated TAO [134]. Interestingly, Mukai et al. showed that none of the polymorphisms in the IL-18 gene were associated with GD, but the CC genotype and C allele frequencies of the IL-18 gene G-137C polymorphism were greater in TAO patients than in patients without evident ophthalmopathy [135]. Subsequently, IL-18 was found to be significantly elevated in the tears of TAO patients [136], which demonstrated that it participated in the pathogenesis of TAO. There are few studies on IL-18 in TAO, and more research is needed in the future to elucidate its role.

### 2.6. Chemokines in TAO

Chemokines are signaling proteins that induce directed chemotaxis in responsive cells and exert biological effects through interactions with G-protein-linked transmembrane receptors on their target cells [137]. Chemokines are small molecules (8-12 kDa), a subfamily of cytokines responsible for immune cell trafficking and lymphoid tissue development, which can be classified into four main classes, namely, C, CC, CXC, and CX3C chemokines [138]. The Th1 response is prevalent in active TAO or GD; and chemokine (C-X-C motif) receptor 3 (CXCR3) and Th1 chemokine (C-X-C motif) ligand 9, 10, and 11 (CXCL9, CXCL10, and CXCL11) are crucial in this process [139]. A study showed that among TAO patients, circulating CXCL10 levels (sCXCL10) were significantly higher in active TAO than those in inactive disease, and stimulation with IFN-*γ* or TNF-*α* plus IFN-*γ* induced CXCL10 release, while CXCL10 production was absent under basal conditions in primary cultures of retrobulbar fibroblasts and retrobulbar preadipocytes from TAO patients, which suggested that fibroblasts or preadipocytes could participate in the pathogenesis of TAO by chemokines [140]. These results were confirmed by another study conducted by Dong et al. [141], which reported that GD patients with ophthalmopathy showed higher sCXCL10 levels than those in patients without ophthalmopathy. Treatment with corticosteroids and teleradiotherapy could significantly reduce CXCL9 and CXCL10 serum concentrations in TAO patients compared to the control group and basal values of TAO patients, which suggests that CXCL9/CXCL10 can partially reflect the activity of orbital inflammation [142, 143]. Regulated upon activation normal T-cell expressed and secreted factor (RANTES/CCL5) belongs to the CC chemokine subfamily and is a target gene of nuclear factor kappa-light platelets [144, 145]. Wan et al. found that IL-17A promotes RANTES expression via the CD40-CD40L combination in orbital fibroblasts in TAO, which further demonstrated the role of chemokines in TAO. Another study showed that aberrant CXCL13/CXCR5 may contribute to deficits in B-lymphocyte homeostasis and result in active TAO [146]. Human primary cell cultures of fibroblasts or preadipocytes in TAO patients differentially secrete CXCL8 and CXCL10, which reflect different phases of the disease; CXCL10 represents the initial phase of disease when IFN-*γ* is preponderant, while CXCL8 represents the later chronic phase of the disease when TNF-*α* is dominant [147].

## 3. Conclusion

TAO is a thyroid organ-specific autoimmune disease, the pathogenesis of which is unclear and still needs to be explored. Increased intraorbital connective and adipose tissue in patients with active TAO is responsible for most orbital complications. The immunological process, especially the role of cytokines, is the key to TAO onset and is the main research direction of current drug therapies. We provide an exhaustive review of the available studies on the cytokines of TAO. Considering the roles of different cytokines in the development of TAO, a solid basis of data from in vivo and in vitro experiments now supports the therapeutic effects. However, there are still some limits in targeted cytokine therapy. First, interventions for targeted cytokine therapy must not only focus on mechanistic advantages but also on the potential risks, such as the side effects, of targeted drugs. Second, the reasons for TAO are miscellaneous, and some studies based on epidemiological results are limited and have only discussed the relationship between cytokines and symptoms of TAO. Thus, further studies, especially human studies, are necessary and important to elucidate the different roles of different cytokines in TAO development and tap their targeted therapeutic potential.

## Figures and Tables

**Table 1:** TSLP (thymic stromal lymphopoietin).

Cell type	Cytokines	Ref.
T lymphocytes		[148–152]
Th1	IL-2, IFN-*γ*, and TNF-*β*	
Th2	IL-4, IL-5, IL-6, IL-9, IL-10, and IL-13	
Th17	IL-17A, IL-17F, and IL-22	
T-regulatory	TGF-*β*, IL-10	
Monocytes	IL-1*α*, IL-1*β*, IL-6, IL-10, and TNF-*α*	[153, 154]
NK cells	IL-5, IL-10, IL-13, IL-17A, IL-22, TNF-*α*, and IFN-*γ*	[155]
B lymphocytes		[156]
Regulatory B cells	IL-10, TGF-*β*1	
Effector B cells	IL-2, IL-4, IL-6, and TNF-*α* (Be-2 cells) IL-12, IFN-*γ*, and TNF-*α* (Be-1 cells)	
Mast cell	IL-4, IL-13	[157]
Fibroblasts	TGF-*β*, IL-16, RANTES, IL-1*α*, IL-1*β*, IL-6, and IL-8	[18, 66, 123, 158]
Macrophages	IL-1*α*, IL-1Ra, IL-1*β*, IL-6, IL-10, IL-12, IL-23,	[159, 160]
	TGF-*β*, TNF-*α*	
Dendritic cells	IL-1, IL-4, IL-6, IL-10, IL-12, IL-15, IL-17, IL-23,	[161, 162]
	and TNF-*α*	
Granulocytes	IL-1*α*, IL-1*β*, IL-4, IL-13, MIP-1*α*, IL-27, IL-17, and IFN-*γ*	[163–165]
Epithelial cells	IL-25, IL-33, and TSLP	[166]
Endothelial cells	IL-1, IL-8, MCP-1, and IFN-*α*	[167]

## Data Availability

The datasets of the current study are available from the corresponding author on reasonable request.
